# Quantum Contextual Advantage Depending on Nonzero Prior Probabilities in State Discrimination of Mixed Qubit States

**DOI:** 10.3390/e23121583

**Published:** 2021-11-26

**Authors:** Jaehee Shin, Donghoon Ha, Younghun Kwon

**Affiliations:** 1 Department of Applied Physics, Center for Bionano Intelligence Education and Research, Hanyang University (ERICA), Ansan 15588, Korea; physics@hanyang.ac.kr; 2 Department of Applied Mathematics and Institute of Natural Sciences, Kyung Hee University, Yongin 17104, Korea; donghoon@khu.ac.kr

**Keywords:** contextuality, ontological model, minimum error discrimination

## Abstract

Recently, Schmid and Spekkens studied the quantum contextuality in terms of state discrimination. By dealing with the minimum error discrimination of two quantum states with identical prior probabilities, they reported that quantum contextual advantage exists. Meanwhile, if one notes a striking observation that the selection of prior probability can affect the quantum properties of the system, it is necessary to verify whether the quantum contextual advantage depends on the prior probabilities of the given states. In this paper, we consider the minimum error discrimination of two states with arbitrary prior probabilities, in which both states are pure or mixed. We show that the quantum contextual advantage in state discrimination may depend on the prior probabilities of the given states. In particular, even though the quantum contextual advantage always exists in the state discrimination of two nonorthogonal pure states with nonzero prior probabilities, the quantum contextual advantage depends on prior probabilities in the state discrimination of two mixed states.

## 1. Introduction

Quantum contextuality is an essential concept that reveals the nonclassicality of quantum mechanics. Kochen and Specker [1] proved that quantum mechanics could not be described using a deterministic hidden variable model independent of the measurement. Later, Spekkens [2] defined noncontextuality by introducing a noncontextual ontological model, which is based on an operational theory.

State discrimination is to figure out what the given state is. The problem can be clarified as follows. First, a sender prepares a state with a specific prior probability. Next, the sender sends the state to the receiver, and the receiver determines what the state is. In the state discrimination, there are many strategies such as minimum error discrimination (MED) [3,4,5,6,7,8,9,10,11,12,13,14,15], unambiguous discrimination [16,17,18,19,20,21,22,23], discrimination with a fixed error [24,25,26,27] etc. In particular, the MED is the optimal measurement strategy that minimizes the average error probability. In the MED, a general solution exists for two quantum states [3] but a general solution to more than two quantum states does not exist. Nevertheless, state discrimination is used in wide application of quantum information processing [28,29,30,31,32,33,34].

Schmid and Spekkens [35] investigated noncontextuality in terms of state discrimination. They studied noncontextuality by considering the minimum error discrimination of two nonorthogonal pure quantum states with identical prior probabilities. They found that the maximum success probability(guessing probability) of the minimum error discrimination in the noncontextual model is lower than that in the quantum model. In other words, quantum contextual advantage, which the preparation-noncontextual model cannot achieve, exists in the MED of two pure qubit states with identical prior probabilities. Therefore, state discrimination may be a valuable tool for quantum contextual advantage that noncontextual ontological models cannot achieve.

According to a recent investigation, the selection of prior probability can affect the quantum properties of the system [36,37]. Therefore, it is necessary to verify whether the quantum contextual advantage depends on the prior probabilities of the given states when the MED of two pure quantum states with arbitrary prior probabilities is considered. Furthermore, it should be determined whether the quantum contextual advantage occurs in the MED of two mixed quantum states with arbitrary prior probabilities.

Therefore, in this study, we investigated the dependence of quantum contextual advantage on the prior probabilities of the given states by considering the MED of two pure(mixed) quantum states with arbitrary prior probabilities. As a result, we showed that the quantum contextual advantage exists regardless of nonzero prior probabilities in MED of two nonorthogonal pure qubit states. However, we observed that in the MED of two nonorthogonal mixed qubit states, the quantum contextual advantage depends on nonzero prior probabilities.

## 2. Preliminaries

### 2.1. Quantum Theory and Discrimination between Two Mixed Qubit States

In the quantum theory, a state of a two-level system(or qubit state) is expressed by a density operator in two-dimensional complex Hilbert space H. A measurement performed on the qubit system is expressed using a positive operator-valued measure(POVM) that consists of positive semidefinite operators $M_{i}$ on H satisfying $\sum_{i}M_{i}=𝟙$. Here, 𝟙 is the identity operator on H. In addition, when a measurement ${\left\{M_{i}\right\}}_{i}$ is performed on qubit state ρ prepared in the qubit system, the probability of obtaining the measurement outcome corresponding to $M_{i}$ is expressed as $\mathrm{Tr}(\rho M_{i})$, according to Born’s rule.

Now, let us consider the case where using measurement $\left\{M_{1},M_{2}\right\}$, one discriminates two qubit states $\rho_{1}$ and $\rho_{2}$ of the qubit state ensemble ${\left\{\eta_{i},\rho_{i}\right\}}_{i=1}^{2}$,

(1) $$\begin{array}{ccc} \eta_{1}=\frac{1}{2}\left(1+\sqrt{1-r}\right), & \rho_{1}=\epsilon \psi_{1}+\left(1-\epsilon \right)\frac{𝟙}{2}, & 0<r\leq 1,\\ \eta_{2}=\frac{1}{2}\left(1-\sqrt{1-r}\right), & \rho_{2}=\epsilon \psi_{2}+\left(1-\epsilon \right)\frac{𝟙}{2}, & 0<\epsilon \leq 1, \end{array}$$

where $\eta_{i}$ is the probability that the qubit state $\rho_{i}$ is prepared and $\psi_{i}$ is a pure qubit state satisfying $0<\mathrm{Tr}(\psi_{1}\psi_{2})<1$. Please note that the mixed states $\rho_{1}$ and $\rho_{2}$, which will be used throughout this paper, can be understood as the outputs of the quantum channel with white noise for the state inputs $\psi_{1}$ and $\psi_{2}$. When $M_{i}$ is a measurement for detecting $\rho_{i}$, the probability that the given state can be correctly guessed becomes

(2) $$\begin{array}{c} p_{s}^{Q}\left({\left\{\eta_{i},\rho_{i}\right\}}_{i=1}^{2}\right)=\eta_{1}\mathrm{Tr}\left(\rho_{1}M_{1}\right)+\eta_{2}\mathrm{Tr}\left(\rho_{2}M_{2}\right). \end{array}$$

The MED of ${\left\{\eta_{i},\rho_{i}\right\}}_{i=1}^{2}$ is to maximize $p_{s}^{Q}\left({\left\{\eta_{i},\rho_{i}\right\}}_{i=1}^{2}\right)$. Based on the Helstrom bound [3], the maximum of $p_{s}^{Q}\left({\left\{\eta_{i},\rho_{i}\right\}}_{i=1}^{2}\right)$ can be expressed as follows:

(3) $$\begin{array}{c} p_{\max}^{Q}\left({\left\{\eta_{i},\rho_{i}\right\}}_{i=1}^{2}\right)=\frac{1}{2}\left(1+\mathrm{Tr}\right|\eta_{1}\rho_{1}-\eta_{2}\rho_{2}\left|\right)=\left\{\begin{array}{ccc} \eta_{1} & \mathrm{for} & 0<r\leq r_{Q},\\ p_{Q} & \mathrm{for} & r_{Q}<r\leq 1, \end{array}\right. \end{array}$$

where

(4) $$\begin{array}{c} c_{Q}=\mathrm{Tr}\left(\psi_{1}\psi_{2}\right),\;r_{Q}=\frac{1-\epsilon^{2}}{1-c_{Q}\epsilon^{2}},\;p_{Q}=\frac{1}{2}\left(1+\epsilon \sqrt{1-c_{Q}r}\right). \end{array}$$

In the region of $0<r\leq r_{Q}$, the optimal success probability $p_{\max}^{Q}\left({\left\{\eta_{i},\rho_{i}\right\}}_{i=1}^{2}\right)$ can be determined by guessing the given state as $\rho_{1}$ without a measurement [38]. However, within the region of $r_{Q}<r\leq 1$, $p_{\max}^{Q}\left({\left\{\eta_{i},\rho_{i}\right\}}_{i=1}^{2}\right)$ cannot be obtained without measurements. The optimal measurement consists of two orthogonal rank-one projectors mapping onto eigenspaces of $\eta_{1}\rho_{1}-\eta_{2}\rho_{2}$ [3].

### 2.2. Operational Theory and Preparation-Noncontextual Ontological Model

Let us understand the quantum theory from the perspective of operational theory, to explain preparations and measurements through primitive laboratory operations. In the operational theory, when every measurement M to two preparations P and $P^{\prime }$ provides the identical statistics, P and $P^{\prime }$ are operationally equivalent [2,35], i.e.,

(5) $$\begin{array}{ccc} P\left(k|M,P\right)=P\left(k|M,P^{\prime }\right)\;\forall M\;\forall k\; & \Rightarrow & P≃P^{\prime }, \end{array}$$

where P(k|M,P) is the probability that the measurement outcome is *k* when measurement M is performed on preparation P. We use $P_{\rho }$ to represent the preparation of a quantum system corresponding to a density operator ρ. Therefore, every preparation of a quantum system, expressed by an identical density operator, is operationally equivalent.

Now, let us briefly explain the ontological model of the operational theory. In the operational theory, every system of an ontological model has an ontic state space Λ revealing its physical properties. Furthermore, the preparation P and measurement M of the system are described by the *epistemic state*$\mu_{P}$ and ${\left\{\xi_{k|M}\right\}}_{k}$ being a set of *response functions*, which satisfy the following relations:

(6) $$\begin{array}{cc} \mu_{P}\left(\lambda \right)\geq 0\;\forall \lambda , & \int_{\Lambda }\mu_{P}\left(\lambda \right)d\lambda =1,\\ \xi_{k|M}\left(\lambda \right)\geq 0\;\forall \lambda \;\forall k, & \sum_{k}\xi_{k|M}\left(\lambda \right)=1\;\forall \lambda . \end{array}$$

The probability that the measurement outcome is *k*, when measurement M is performed on preparation P, is expressed as follows:

(7) $$\begin{array}{c} P\left(k|M,P\right)=\int_{\Lambda }\xi_{k|M}\left(\lambda \right)\mu_{P}\left(\lambda \right)d\lambda , \end{array}$$

where $\mu_{\rho }$ and $\xi_{k|M}$ are the epistemic state and the response function, respectively, corresponding to preparation $P_{\rho }$ and measurement M. We use $\left\{\xi_{\psi_{i}|B_{i}},\xi_{\psi_{i}^{⊥}|B_{i}}\right\}$ to describe the response functions corresponding to the measurement $B_{i}:=\left\{\psi_{i},\psi_{i}^{⊥}\right\}$, where $\psi_{i}^{⊥}$ is the pure qubit state orthogonal to $\psi_{i}$. If experiments of state preparations $\psi_{i}$,$\psi_{i}^{⊥}$, and measurements $B_{i}$ are expressed by an ontological model, the model should produce the following relations:

(8) $$\begin{array}{ccc} \int_{\Lambda }\xi_{\psi_{i}|B_{i}}\left(\lambda \right)\mu_{\psi_{i}}\left(\lambda \right) & = & \mathrm{Tr}(\psi_{i}\psi_{i})=1\;\;\forall i,\\ \int_{\Lambda }\xi_{\psi_{i}|B_{i}}\left(\lambda \right)\mu_{\psi_{i}^{⊥}}\left(\lambda \right) & = & \mathrm{Tr}(\psi_{i}^{⊥}\psi_{i})=0\;\;\forall i,\\ \int_{\Lambda }\xi_{\psi_{j}|B_{j}}\left(\lambda \right)\mu_{\psi_{i}}\left(\lambda \right) & = & \mathrm{Tr}\left(\psi_{i}\psi_{j}\right)=c_{Q}\;\forall i\neq j. \end{array}$$

The ontological model, which assigns an identical epistemic state to two operationally equivalent preparations, is called *preparation noncontextual* [2,35]. In this work, we consider a preparation-noncontextual ontological model to describe the preparation of the system. For instance, the preparation of the mixed qubit state $\rho_{i}$, defined in Equation (1), is operationally equivalent to the preparation of the qubit system where pure qubit state $\psi_{i}$ and maximally mixed state $\frac{𝟙}{2}$ are prepared with the probabilities of ϵ and 1−ϵ, respectively. Therefore, the preparation noncontextuality implies that

(9) $$\begin{array}{c} \mu_{\rho_{1}}=\epsilon \mu_{\psi_{1}}+\left(1-\epsilon \right)\mu_{\frac{𝟙}{2}},\;\mu_{\rho_{2}}=\epsilon \mu_{\psi_{2}}+\left(1-\epsilon \right)\mu_{\frac{𝟙}{2}}. \end{array}$$

As another example, preparation $P_{\frac{𝟙}{2}}$ of the maximally mixed state $\frac{𝟙}{2}$ is operationally equivalent to the preparation of a qubit system where two orthogonal states $\psi_{i}$ and $\psi_{i}^{⊥}$ are prepared with identical probabilities. Then, preparation noncontextuality indicates that

(10) $$\begin{array}{c} \mu_{\frac{𝟙}{2}}=\frac{1}{2}\mu_{\psi_{1}}+\frac{1}{2}\mu_{\psi_{1}^{⊥}}=\frac{1}{2}\mu_{\psi_{2}}+\frac{1}{2}\mu_{\psi_{2}^{⊥}}. \end{array}$$

Because, in preparation-noncontextual model, $\mathrm{supp}(\mu_{\frac{𝟙}{2}})$ is Λ, from Equations (8) and (10) we can obtain the following relations [2,35]:

(11) $$\begin{array}{cc} \mathrm{supp}\left(\mu_{\psi_{1}}\right)⋃\mathrm{supp}\left(\mu_{\psi_{1}^{⊥}}\right)=\Lambda , & \xi_{\psi_{1}|B_{1}}\left(\lambda \right)=\left\{\begin{array}{cc} 1, & \lambda \in \mathrm{supp}(\mu_{\psi_{1}}),\\ 0, & \lambda \in \mathrm{supp}(\mu_{\psi_{1}^{⊥}}), \end{array}\right.\\ \mathrm{supp}\left(\mu_{\psi_{2}}\right)⋃\mathrm{supp}\left(\mu_{\psi_{2}^{⊥}}\right)=\Lambda , & \xi_{\psi_{2}|B_{2}}\left(\lambda \right)=\left\{\begin{array}{cc} 1, & \lambda \in \mathrm{supp}(\mu_{\psi_{2}}),\\ 0, & \lambda \in \mathrm{supp}(\mu_{\psi_{2}^{⊥}}), \end{array}\right. \end{array}$$

where $\mathrm{supp}(\mu_{\rho })$ and $\mathrm{supp}(\xi_{k|M})$ are the supports of the epistemic state $\mu_{\rho }$ and response function $\xi_{k|M}$, respectively, i.e.,

(12) $$\mathrm{supp}\left(\mu_{\rho }\right)=\left\{\lambda \in \Lambda \;|\;\mu_{\rho }\left(\lambda \right)>0\right\}.$$

Considering outcome-determinism of Equation (11), we obtain

(13) $$\begin{array}{c} \int_{\Lambda }\xi_{\psi_{2}|B_{2}}\left(\lambda \right)\mu_{\psi_{1}}\left(\lambda \right)d\lambda =\int_{\Lambda }\xi_{\psi_{1}|B_{1}}\left(\lambda \right)\mu_{\psi_{2}}\left(\lambda \right)d\lambda =c_{Q},\\ \int_{\Lambda }\xi_{\psi_{2}^{⊥}|B_{2}}\left(\lambda \right)\mu_{\psi_{1}}\left(\lambda \right)d\lambda =\int_{\Lambda }\xi_{\psi_{1}^{⊥}|B_{1}}\left(\lambda \right)\mu_{\psi_{2}}\left(\lambda \right)d\lambda =1-c_{Q}. \end{array}$$

Equation (13) represents the expressions to ideal confusability of two pure states in the preparation-noncontextual model.

Equations (10) and (13) hold in two arbitrary states $\psi_{1}$ and $\psi_{2}$. Therefore, the preparation-noncontextual model of two pure orthogonal qubit states ϕ and $\phi^{⊥}$, which describe experiments to state preparations ϕ and $\phi^{⊥}$ and measurement $M=\{\phi ,\phi^{⊥}\}$, should produce the following relations:

(14) $$\begin{array}{c} \int_{\Lambda }\xi_{\phi |M}\left(\lambda \right)\mu_{\frac{𝟙}{2}}\left(\lambda \right)d\lambda =\int_{\Lambda }\xi_{\phi^{⊥}|M}\left(\lambda \right)\mu_{\frac{𝟙}{2}}\left(\lambda \right)d\lambda =\frac{1}{2}. \end{array}$$

If $p_{\max}^{Q}\left({\left\{\eta_{i},\rho_{i}\right\}}_{i=1}^{2}\right)>\eta_{1}$ in the MED of ${\left\{\eta_{i},\rho_{i}\right\}}_{i=1}^{2}$, the optimal measurement is rank-one projective. Therefore, if the maximum success probability achievable in the preparation-noncontextual model is higher than $\eta_{1}$, the measurement that provides the maximum success probability satisfies Equation (14), i.e.,

(15) $$\begin{array}{c} \int_{\Lambda }\xi_{k|M^{⋆}}\left(\lambda \right)\mu_{\frac{𝟙}{2}}\left(\lambda \right)d\lambda =\frac{1}{2}\;\forall k\in \left\{1,2\right\}\;\;if\;\;\sum_{i=1}^{2}\eta_{i}\int_{\Lambda }\xi_{i|M^{⋆}}\left(\lambda \right)\mu_{\rho_{i}}\left(\lambda \right)d\lambda >\eta_{1}, \end{array}$$

where $M^{⋆}$ is the measurement providing the maximum of $\sum_{i=1}^{2}\eta_{i}\int_{\Lambda }\xi_{i|M}\left(\lambda \right)\mu_{\rho_{i}}\left(\lambda \right)d\lambda$ over all possible measurements M with two outcomes k∈{1,2}.

## 3. Results

In this section, we consider the preparation-noncontextual model that reproduces the statistics of Equations (8) and (15).

First, let us consider a case discriminating two preparations $P_{\psi_{1}}$ and $P_{\psi_{2}}$ provided by probabilities of $\eta_{1}$ and $\eta_{2}$, respectively, using measurement M with two outcomes k∈{1,2}. When the outcome *k* of M indicates the detection of $P_{\psi_{k}}$, the probability that the given preparation is guessed correctly can be expressed as follows:

(16) $$\begin{array}{ccc} p_{s}^{\mathrm{NC}}\left({\left\{\eta_{i},P_{\psi_{i}}\right\}}_{i=1}^{2}\right) & = & \sum_{i=1}^{2}\eta_{i}\int_{\Lambda }\xi_{i|M}\left(\lambda \right)\mu_{\psi_{i}}\left(\lambda \right)d\lambda \\ \; & = & \frac{1}{2}+\frac{1}{2}\int_{\Lambda }\left(\eta_{1}\mu_{\psi_{1}}\left(\lambda \right)-\eta_{2}\mu_{\psi_{2}}\left(\lambda \right)\right)\left(\xi_{1|M}\left(\lambda \right)-\xi_{2|M}\left(\lambda \right)\right)d\lambda , \end{array}$$

where the second equality is obtained using the relation of $\eta_{1}+\eta_{2}=1$ and the constraints of the response functions on Equation (6). Let the maximum of $p_{s}^{\mathrm{NC}}\left({\left\{\eta_{i},P_{\psi_{i}}\right\}}_{i=1}^{2}\right)$ be denoted as $p_{\max}^{\mathrm{NC}}\left({\left\{\eta_{i},P_{\psi_{i}}\right\}}_{i=1}^{2}\right)$. The following lemma provides the upper bound of $p_{\max}^{\mathrm{NC}}\left({\left\{\eta_{i},P_{\psi_{i}}\right\}}_{i=1}^{2}\right)$.

**Lemma** **1.** *$p_{\max}^{\mathrm{NC}}\left({\left\{\eta_{i},P_{\psi_{i}}\right\}}_{i=1}^{2}\right)$ has an upper bound, expressed as follows:*

(17) $$p_{\max}^{\mathrm{NC}}\left({\left\{\eta_{i},P_{\psi_{i}}\right\}}_{i=1}^{2}\right)\leq 1-\eta_{2}c_{Q}.$$

**Proof.**  Suppose that M is the measurement providing $p_{\max}^{\mathrm{NC}}\left({\left\{\eta_{i},P_{\psi_{i}}\right\}}_{i=1}^{2}\right)$. For any ontic state λ∈Λ, the inequality of $|\xi_{1|M}\left(\lambda \right)-\xi_{2|M}\left(\lambda \right)|\leq 1$ holds and using Equation (16) we obtain the following inequalities:

(18) $$\begin{array}{ccc} 2p_{\max}^{\mathrm{NC}}\left({\left\{\eta_{i},P_{\psi_{i}}\right\}}_{i=1}^{2}\right)-1\; & \leq & \int_{\Lambda }|\eta_{1}\mu_{\psi_{1}}\left(\lambda \right)-\eta_{2}\mu_{\psi_{2}}\left(\lambda \right)|d\lambda \\ \; & = & \int_{\mathrm{supp}\left(\mu_{\psi_{1}}\right)\cap \mathrm{supp}\left(\mu_{\psi_{2}}\right)}|\eta_{1}\mu_{\psi_{1}}\left(\lambda \right)-\eta_{2}\mu_{\psi_{2}}\left(\lambda \right)|d\lambda \\ \; & \; & +\int_{\mathrm{supp}\left(\mu_{\psi_{1}}\right)\cap \mathrm{supp}\left(\mu_{\psi_{2}^{⊥}}\right)}|\eta_{1}\mu_{\psi_{1}}\left(\lambda \right)-\eta_{2}\mu_{\psi_{2}}\left(\lambda \right)|d\lambda \\ \; & \; & +\int_{\mathrm{supp}\left(\mu_{\psi_{1}^{⊥}}\right)\cap \mathrm{supp}\left(\mu_{\psi_{2}}\right)}|\eta_{1}\mu_{\psi_{1}}\left(\lambda \right)-\eta_{2}\mu_{\psi_{2}}\left(\lambda \right)|d\lambda \\ \; & \; & +\int_{\mathrm{supp}\left(\mu_{\psi_{1}^{⊥}}\right)\cap \mathrm{supp}\left(\mu_{\psi_{2}^{⊥}}\right)}|\eta_{1}\mu_{\psi_{1}}\left(\lambda \right)-\eta_{2}\mu_{\psi_{2}}\left(\lambda \right)|d\lambda \\ \; & = & \left(\eta_{1}-\eta_{2}\right)\int_{\mathrm{supp}\left(\mu_{\psi_{1}}\right)\cap \mathrm{supp}\left(\mu_{\psi_{2}}\right)}\mu_{\psi_{1}}\left(\lambda \right)d\lambda \\ \; & \; & +\eta_{1}\int_{\mathrm{supp}\left(\mu_{\psi_{1}}\right)\cap \mathrm{supp}\left(\mu_{\psi_{2}^{⊥}}\right)}\mu_{\psi_{1}}\left(\lambda \right)d\lambda \\ \; & \; & +\eta_{2}\int_{\mathrm{supp}\left(\mu_{\psi_{1}^{⊥}}\right)\cap \mathrm{supp}\left(\mu_{\psi_{2}}\right)}\mu_{\psi_{2}}\left(\lambda \right)d\lambda \\ \; & = & \left(\eta_{1}-\eta_{2}\right)\int_{\Lambda }\xi_{\psi_{2}|B_{2}}\left(\lambda \right)\mu_{\psi_{1}}\left(\lambda \right)d\lambda \\ \; & \; & +\eta_{1}\int_{\Lambda }\xi_{\psi_{2}^{⊥}|B_{2}}\left(\lambda \right)\mu_{\psi_{1}}\left(\lambda \right)d\lambda +\eta_{2}\int_{\Lambda }\xi_{\psi_{1}^{⊥}|B_{1}}\left(\lambda \right)\mu_{\psi_{2}}\left(\lambda \right)d\lambda \\ \; & = & 1-2\eta_{2}c_{Q}, \end{array}$$

where the first three equalities are derived using preparation noncontextuality and Equation (11), and the final equality is obtained by Equation (13). Therefore, the above inequality (17) holds. □

Now, let us investigate the relationship between $p_{\max}^{\mathrm{NC}}\left({\left\{\eta_{i},P_{\psi_{i}}\right\}}_{i=1}^{2}\right)$ and $p_{\max}^{Q}\left({\left\{\eta_{i},\psi_{i}\right\}}_{i=1}^{2}\right)$. It may be observed that within the region of $0<c_{Q}<1$, a nonzero gap between $p_{\max}^{\mathrm{NC}}\left({\left\{\eta_{i},P_{\psi_{i}}\right\}}_{i=1}^{2}\right)$ and $p_{\max}^{Q}\left({\left\{\eta_{i},\psi_{i}\right\}}_{i=1}^{2}\right)$ exists, as follows:

(19) $$\begin{array}{c} p_{\max}^{\mathrm{NC}}\left({\left\{\eta_{i},P_{\psi_{i}}\right\}}_{i=1}^{2}\right)\leq 1-\eta_{2}c_{Q}<\frac{1}{2}\left(1+\sqrt{1-c_{Q}r}\right)=p_{\max}^{Q}\left({\left\{\eta_{i},\psi_{i}\right\}}_{i=1}^{2}\right), \end{array}$$

where the inequality holds because of Lemma 1 and the equality holds because of the Helstrom bound of Equation (3). Therefore, in the case of discrimination of two pure qubit states such as ${\left\{\eta_{i},\psi_{i}\right\}}_{i=1}^{2}$, the quantum contextual advantage, which means that $p_{\max}^{Q}\left({\left\{\eta_{i},\psi_{i}\right\}}_{i=1}^{2}\right)$ is higher than $p_{\max}^{\mathrm{NC}}\left({\left\{\eta_{i},P_{\psi_{i}}\right\}}_{i=1}^{2}\right)$, exists regardless of nonzero prior probabilities. The following theorem summarizes the result.

**Theorem** **1.** *For the MED of two pure qubit states $\psi_{1}$ and $\psi_{2}$ with $0<\mathrm{Tr}(\psi_{1}\psi_{2})<1$, the quantum contextual advantage exists regardless of the nonzero prior probabilities of $\psi_{1}$ and $\psi_{2}$.*

Let us now consider a situation discriminating two preparations $P_{\rho_{1}}$ and $P_{\rho_{2}}$ given by probabilities of $\eta_{1}$ and $\eta_{2}$ using measurement M with two outcomes k∈{1,2}. When the outcome *k* of M indicates the detection of $P_{\rho_{k}}$, the probability that the given preparation is guessed correctly can be expressed as follows:

(20) $$\begin{array}{ccc} p_{s}^{\mathrm{NC}}\left({\left\{\eta_{i},P_{\rho_{i}}\right\}}_{i=1}^{2}\right) & = & \sum_{i=1}^{2}\eta_{i}\int_{\Lambda }\xi_{i|M}\left(\lambda \right)\mu_{\rho_{i}}\left(\lambda \right)d\lambda \\ \; & = & \epsilon p_{s}^{\mathrm{NC}}\left({\left\{\eta_{i},P_{\psi_{i}}\right\}}_{i=1}^{2}\right)+\left(1-\epsilon \right)\sum_{i=1}^{2}\eta_{i}\int_{\Lambda }\xi_{i|M}\left(\lambda \right)\mu_{\frac{𝟙}{2}}\left(\lambda \right)d\lambda , \end{array}$$

where the second equality holds using Equation (9). Let us denote the maximum of $p_{s}^{\mathrm{NC}}\left({\left\{\eta_{i},P_{\rho_{i}}\right\}}_{i=1}^{2}\right)$ as $p_{\max}^{\mathrm{NC}}\left({\left\{\eta_{i},P_{\rho_{i}}\right\}}_{i=1}^{2}\right)$. The following lemma provides an upper bound for $p_{\max}^{\mathrm{NC}}\left({\left\{\eta_{i},P_{\rho_{i}}\right\}}_{i=1}^{2}\right)$.

**Lemma** **2.** *When $p_{\max}^{\mathrm{NC}}\left({\left\{\eta_{i},P_{\rho_{i}}\right\}}_{i=1}^{2}\right)>\eta_{1}$, $p_{\max}^{\mathrm{NC}}\left({\left\{\eta_{i},P_{\rho_{i}}\right\}}_{i=1}^{2}\right)$ has the following upper bound:*

(21) $$p_{\max}^{\mathrm{NC}}\left({\left\{\eta_{i},P_{\rho_{i}}\right\}}_{i=1}^{2}\right)\leq p_{\mathrm{NC}},$$

*where*

(22) $$\begin{array}{c} p_{\mathrm{NC}}=\frac{1}{2}+\frac{\epsilon }{2}-\eta_{2}c_{Q}\epsilon . \end{array}$$

**Proof.**  Suppose that M is a measurement providing $p_{\max}^{\mathrm{NC}}\left({\left\{\eta_{i},P_{\rho_{i}}\right\}}_{i=1}^{2}\right)$. From Equation (20) and Lemma 1, we can obtain the following inequality:

(23) $$\begin{array}{ccc} p_{\max}^{\mathrm{NC}}\left({\left\{\eta_{i},P_{\rho_{i}}\right\}}_{i=1}^{2}\right)\; & \leq & \epsilon \left(1-\eta_{2}c_{Q}\right)+\left(1-\epsilon \right)\sum_{i=1}^{2}\eta_{i}\int_{\Lambda }\xi_{i|M}\left(\lambda \right)\mu_{\frac{𝟙}{2}}\left(\lambda \right)d\lambda \\ \; & = & \epsilon \left(1-\eta_{2}c_{Q}\right)+\frac{1}{2}\left(1-\eta_{1}\epsilon -\eta_{2}\epsilon \right)=p_{\mathrm{NC}}, \end{array}$$

where the first equality is obtained by Equation (15). Therefore, we can see that Equation (21) holds. □

We can see that $p_{Q}$ defined in Equation (4) is higher than $p_{\mathrm{NC}}$ defined in Equation (22), that is, $p_{Q}>p_{\mathrm{NC}}$. In addition, it should be noted that when one guesses the given preparation as $P_{\rho_{1}}$ without measurement, the success probability becomes the prior probability $\eta_{1}$ and we can see that $p_{\max}^{\mathrm{NC}}\left({\left\{\eta_{i},P_{\rho_{i}}\right\}}_{i=1}^{2}\right)$ is higher than or equal to $\eta_{1}$, i.e.,

(24) $$p_{\max}^{\mathrm{NC}}\left({\left\{\eta_{i},P_{\rho_{i}}\right\}}_{i=1}^{2}\right)\geq \eta_{1}.$$

When $\eta_{1}$ is equal to or higher than $p_{\mathrm{NC}}$, $p_{\max}^{\mathrm{NC}}\left({\left\{\eta_{i},P_{\rho_{i}}\right\}}_{i=1}^{2}\right)$ becomes $\eta_{1}$ due to Lemma 2 and Equation (24). Then, we can obtain the following relations:

(25) $$\begin{array}{ccc} p_{\max}^{\mathrm{NC}}\left({\left\{\eta_{i},P_{\rho_{i}}\right\}}_{i=1}^{2}\right)=\eta_{1}, & \mathrm{if} & r\leq r_{\mathrm{NC}},\\ p_{\max}^{\mathrm{NC}}\left({\left\{\eta_{i},P_{\rho_{i}}\right\}}_{i=1}^{2}\right)\leq p_{\mathrm{NC}}<p_{Q} & \mathrm{if} & r>r_{\mathrm{NC}}, \end{array}$$

where

(26) $$\begin{array}{c} r_{\mathrm{NC}}=\frac{\left(1-\epsilon \right)\left(1+\epsilon -2\epsilon c_{Q}\right)}{{\left(1-\epsilon c_{Q}\right)}^{2}}. \end{array}$$

$r_{\mathrm{NC}}$ is the boundary between regions of $\eta_{1}\geq p_{\mathrm{NC}}$ and $\eta_{1}<p_{\mathrm{NC}}$. We can easily see that $r_{\mathrm{NC}}$ is always higher than $r_{Q}$, i.e.,

(27) $$r_{Q}<r_{\mathrm{NC}}.$$

**Figure 1:**
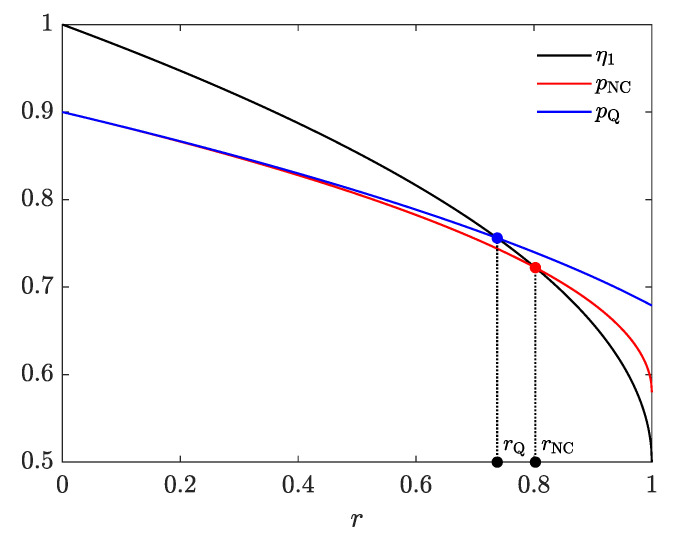
Behavior of $p_{Q}$ and $p_{\mathrm{NC}}$ for $c_{Q}=\epsilon =0.8$. $p_{Q}$(blue) is lower than or equal to $\eta_{1}$(black) for $0<r\leq r_{Q}$, but is higher than $\eta_{1}$ for $r_{Q}<r<1$. $p_{\mathrm{NC}}$(red) is lower than or equal to $\eta_{1}$ for $0<r\leq r_{\mathrm{NC}}$, but is higher than $\eta_{1}$ for $r_{\mathrm{NC}}<r<1$.

Now, let us analyze the property of the quantum contextual advantage in state discrimination of two mixed qubit states, in terms of *r*. First, we can see that in the interval of $r_{Q}<r\leq 1$, it holds that

(28) $$p_{\max}^{\mathrm{NC}}\left({\left\{\eta_{i},P_{\rho_{i}}\right\}}_{i=1}^{2}\right)<p_{\max}^{Q}\left({\left\{\eta_{i},\rho_{i}\right\}}_{i=1}^{2}\right),$$

and the quantum contextual advantage occurs. However, we can observe that in the region of $0<r\leq r_{Q}$, it holds that

(29) $$p_{\max}^{\mathrm{NC}}\left({\left\{\eta_{i},P_{\rho_{i}}\right\}}_{i=1}^{2}\right)=p_{\max}^{Q}\left({\left\{\eta_{i},\rho_{i}\right\}}_{i=1}^{2}\right)=\eta_{1},$$

and the quantum contextual advantage does not exist. This implies that in 0<ϵ<1, the quantum contextual advantage depends on nonzero prior probabilities of two mixed qubit states. Figure 1 shows the behavior of $p_{Q}$ and $p_{\mathrm{NC}}$ in terms of r∈(0,1) at $c_{Q}=\epsilon =0.8$. In the Figure 1, we can see that $p_{Q}$ is lower than or equal to $\eta_{1}$ for $0<r\leq r_{Q}$, but is higher than $\eta_{1}$ for $r_{Q}<r<1$. Moreover, we can observe that $p_{\mathrm{NC}}$ is lower than or equal to $\eta_{1}$ for $0<r\leq r_{\mathrm{NC}}$, but is higher than $\eta_{1}$ for $r_{\mathrm{NC}}<r<1$. The following theorem summarizes our results.

**Theorem** **2.** *For the MED of two mixed qubit states $\rho_{1}$ and $\rho_{2}$ given by Equation (1), the occurrence of quantum contextuality depends on the nonzero prior probabilities of $\rho_{1}$ and $\rho_{2}$.*

## 4. Conclusions

In this work, we investigated quantum contextuality, a critical concept revealing the nonclassicality of quantum mechanics. Noncontextuality was recently studied in terms of state discrimination, which showed that quantum contextual advantage, which a preparation-noncontextual model cannot achieve, exists in the MED of two pure qubit states with identical prior probabilities. However, it should be emphasized that a recent investigation tells us that the selection of prior probability could affect the quantum properties of the system. Therefore, it is necessary to check whether the quantum contextual advantage depends on the prior probabilities of given states.

Therefore, in this study, we analyzed the dependence of the quantum contextual advantage on the prior probabilities of given states by considering the MED of two pure(mixed) quantum states with arbitrary prior probabilities. We found that the quantum contextual advantage occurs regardless of nonzero prior probabilities in MED of two nonorthogonal pure qubit states. However, we observed that the quantum contextual advantage depends on nonzero prior probabilities in MED of two nonorthogonal mixed qubit states. It shows an interesting behavior of the quantum contextual advantage in the MED. Therefore, it should be verified whether quantum contextual advantage exists in other state discrimination types.

## Abbreviations

The following abbreviations are used in this manuscript:

MED	Minimum error discrimination
